# Correction: Al-Harrasi et al. Development and Characterization of Chitosan and Porphyran Based Composite Edible Films Containing Ginger Essential Oil. *Polymers* 2022, *14*, 1782

**DOI:** 10.3390/polym14132518

**Published:** 2022-06-21

**Authors:** Ahmed Al-Harrasi, Saurabh Bhtaia, Mohammed Said Al-Azri, Hafiz A. Makeen, Mohammed Albratty, Hassan A. Alhazmi, Syam Mohan, Ajay Sharma, Tapan Behl

**Affiliations:** 1 Natural & Medical Sciences Research Center, University of Nizwa, P.O. Box 33, Birkat Al Mauz, Nizwa 616, Oman; malazri@unizwa.edu.om; 2 School of Health Science, University of Petroleum and Energy Studies, Dehradun 248007, India; syammohanm@yahoo.com; 3 Pharmacy Practice Research Unit, Clinical Pharmacy Department, College of Pharmacy, Jazan University, Jazan 45142, Saudi Arabia; hafiz@jazanu.edu.sa; 4 Department of Pharmaceutical Chemistry, College of Pharmacy, Jazan University, P.O. Box 114, Jazan 45142, Saudi Arabia; malbratty@jazanu.edu.sa (M.A.); hasalhazmi@gmail.com (H.A.A.); 5 Substance Abuse and Toxicology Research Center, Jazan University, Jazan 45142, Saudi Arabia; 6 Department of Pharmacognosy & Phytochemistry, School of Pharmaceutical Sciences, Delhi Pharmaceutical Sciences and Research University, New Delhi 110017, India; ajaysharmapharma1979@gmail.com; 7 Chitkara College of Pharmacy, Chitkara University, Rajpura 140401, India; tapanbehl31@gmail.com

In the original publication [1], there was a mistake in the top of Figure 5 as published.

Arrows need to be added for tiny particles in (S5).
The yellow arrow in (S2) is not in the correct position.
(S3) and (S4) pictures need to be corrected.

The corrected Figure 5 appears below.

The authors apologize for any inconvenience caused and state that the scientific conclusions are unaffected. This correction was approved by the Academic Editor. The original publication has also been updated.

## Figures and Tables

**Figure 5:**
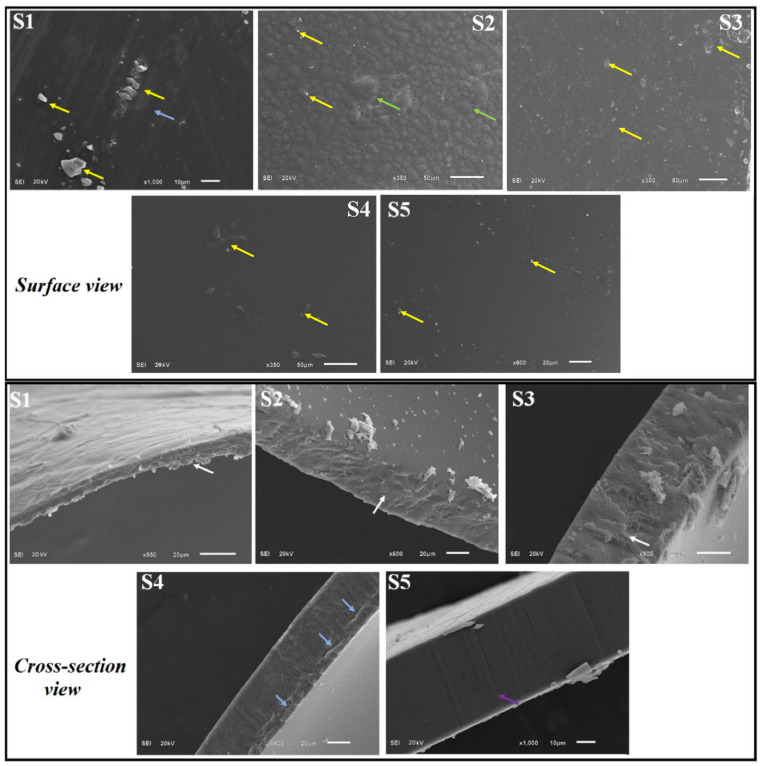
SEM images of CS blank film (S1), CS-Gly film (S2), CS-Gly-GEO film (S3), CS-POR-Gly film (S4), and CS-POR-Gly-GEO film (S5) (superior and cross section view); yellow arrows represent particles over the surface; purple colour arrows indicate compact, uniform, homogenous, and dense structure; green colour represents bulges over the surface and roughness; white arrows represent porosity and irregularity in the film; and blue arrows represents the cracks over the surface and cross-section.
